# Integrated analysis of gene expression changes associated with coronary artery disease

**DOI:** 10.1186/s12944-019-1032-5

**Published:** 2019-04-09

**Authors:** Liu Miao, Rui-Xing Yin, Feng Huang, Shuo Yang, Wu-Xian Chen, Jin-Zhen Wu

**Affiliations:** 1 Department of Cardiology, Institute of Cardiovascular Diseases, The First Affiliated Hospital, Guangxi Medical University, Nanning, 530021 Guangxi People’s Republic of China; 2 Guangxi Key Laboratory Base of Precision Medicine in Cardio-cerebrovascular Disease Control and Prevention, 6 Shuangyong Road, Nanning, 530021 Guangxi People’s Republic of China; 3 Guangxi Clinical Research Center for Cardio-cerebrovascular Diseases, 6 Shuangyong Road, Nanning, 530021 Guangxi People’s Republic of China

**Keywords:** Array data, Gene ontology annotation, Kyoto encyclopedia of genes and genomes (KEGG) pathway, Database for annotation, Visualization and integrated discovery, Protein-protein interaction (PPI) network, Gene expression and cox proportional hazards regression

## Abstract

**Background:**

This study investigated the pathways and genes involved in coronary artery disease (CAD) and the associated mechanisms.

**Methods:**

Two array data sets of GSE19339 and GSE56885 were downloaded. The limma package was used to analyze the differentially expressed genes (DEGs) in normal and CAD specimens. Examination of DEGs through Kyoto Encyclopedia of Genes and Genomes (KEGG) pathway enrichment and Gene Ontology annotation was achieved by Database for Annotation, Visualization and Integrated Discovery (DAVID). The Cytoscape software facilitated the establishment of the protein-protein interaction (PPI) network and Molecular Complex Detection (MCODE) was performed for the significant modules.

**Results:**

We identified 413 DEGs (291 up-regulated and 122 down-regulated). Approximately 256 biological processes, only 1 cellular component, and 21 molecular functions were identified by GO analysis and 10 pathways were enriched by KEGG. Moreover, 264 protein pairs and 64 nodes were visualized by the PPI network. After the MCODE analysis, the top 4 high degree genes, including interleukin 1 beta (*IL1B*, degree = 29), intercellular adhesion molecule 1 (*ICAM1*, degree = 25), Jun proto-oncogene (*JUN*, degree = 23) and C-C motif chemokine ligand 2 (*CCL2*, degree = 20) had been identified to validate in RT-PCR and Cox proportional hazards regression between CAD and normals.

**Conclusions:**

The relative expression of *IL1B*, *ICAM1* and *CCL2* was higher in CAD than in normal controls (*P* < 0.05–0.001), but only *IL1B* and *CCL2* genes were confirmed after testing the gene expression in blood and/or analyzing in Cox proportional hazards regression (*P* < 0.05–0.001), and the proper mechanism may involve in the AGE-RAGE signaling pathway, fluid shear stress, the tumor necrosis factor (TNF) and cytokine-cytokine receptor interaction.

**Electronic supplementary material:**

The online version of this article (10.1186/s12944-019-1032-5) contains supplementary material, which is available to authorized users.

## Background

As the number one cause of mortality, coronary artery disease (CAD) contributes to approximately 17 million deaths every year across the world [1], and almost 700,000 deaths due to CAD are recorded annually in China [2]. Being a complicated and multifactorial condition, CAD results from a variety of environmental exposures and genetic variation, including gender, age, diabetes, hypertension, dyslipidemia, cigarette smoking, and family history [3–7]. To some extent reaching an agreement about the early prevention of CAD would be effective.

As a practical way to identify gene expression changes, a microarray analysis may be a useful method to help in the early diagnosis of CAD [8]. However, numerous previous studies reveal that microarray results are not reproducible or sensitive to the changes in information [9, 10]. Even worse, when over 50 thousand probes in a microarray were used to analyze hundreds of samples, only the inaccuracy of potential predictors was found.

With these situations, an integrated analysis may be used to increase the integrality and reliability of the conclusions. In this way, we wanted to achieve a more precise method of detecting differentially expressed genes, and aimed to find their potential biological functions. The Gene Expression Omnibus (GEO, http://www.ncbi.nlm.nih.gov/geo/) [11] is a global free-access repository of next-generation sequence functional genomic data sets and high-throughput microarray deposited by researchers worldwide. The information in these databases can be freely downloaded in many formats. In the current study, we analyzed two public microarray datasets from the GEO repository to determine the genes that had differential expression in individuals suffering from CAD compared to controls using combined *P* values and we attempted to provide advice on the bio-labelling for on time safeguarding and therapy according to the features of these genes.

## Materials and methods

### Affymetrix microarray data

There were two gene expression profile data sets. GSE19339 was retrieved from GPL570 Affymetrix Human Genome U133 Plus 2.0 array. GSE56885 was derived from the GPL15207 Affymetrix Human Genome array. The present study included 14 samples, which comprised 8 CAD samples and 6 normal/healthy samples. However, the specific results of each sample were not supported. In the current research, all of these samples were selected. The CEL files were transformed into the expression value matrix using the Affy package in R with RMA methods to normalize the expression value matrix [12]. Afterwards, the Bioconductor in R was used to convert the probe data to gene [13]. Any gene that corresponded to multiple probes, the mean expression value of such a gene should be chosen.

### Differentially expressed genes (DEGs) analysis

The limma package [14] in R enabled identification of DEGs based on the comparison between the control and the CAD samples. The threshold values were |log_2_fold-change| > 2 and adjusted *P* < 0.05.

### GO and pathway enrichment analyses

Studies on large-scale transcription data or genomic data were usually performed based on Gene Ontology (GO) analyses [15]. The Kyoto Encyclopedia of Genes and Genomes (KEGG) pathway database harbors information relating to the networks among genes or molecules, which was used for genetic studies [16]. The Database for Annotation, Visualization and Integrated Discovery (DAVID) contained analytical tools and integrated biological information which was used to interpret the functions of large protein or gene pools [17]. Here, DAVID (version 6.8) was used to enrich the GO functions and pathways of specific DEGs in the KEGG (http://www.genome.ad.jp/kegg/) and GO (http://www.geneontology.org) databases and the R package Goplot [18] with an adjusted *P*-value (*q*-value) of < 0.05.

### Construction of PPI interaction network

The Search Tool for the Retrieval of Interacting Genes (STRING V10.5) [19] database (https://string-db.org/) is a useful platform to study the interactions between experimental and predicted proteins. The predictive capacity of STRING is based on text mining, databases, co-expression experiments, co-occurrence, gene fusion and neighborhood which are achieved using the Cytoscape (version 3.60) [20]. Then, a combined score was assigned to the interactions among protein pairs in the database. To examine the key genes in the network and potential PPI correlations, the DEGs were mapped on the data and the cut-off value was set as a combined score of > 0.9 [21]. The significance of protein nodes in the network was described using a degree. All of the three protocols were handled by R software package igraph version 1.0.1 [22].

### Module analysis

Protein networks such as the network module contain useful information regarding the biological functions of biological molecules. The outstanding clustering module was determined using the Cytoscape software package Molecular Complex Detection (MCODE) [23] . Subsequently, the DAVID online tool was used to analyze the KEGG pathway enriched by the DEGs among the modules. Count ≥2 and EASE ≤0.05 were chosen as the cut-off values and MCODE score > 6 as the threshold for the subsequent analysis.

### Study population and follow-up

A total of 206 outpatient were recruited for a complaint of chest pain at the First Affiliated Hospital, Guangxi Medical University from Jan. 1, 2015 to Dec. 31, 2016. A monthly follow-up was performed for patients through a telephone interview and the interviewer was blinded to the genetic status of the patient. The endpoint of the follow-up was a diagnosis of acute coronary symptoms, and cardiac deaths such as deaths due to sudden cardiac death or pump failure. Coronary angiography was performed for patients with suspected CAD or other unrelated conditions where angiographic examination was required. Two experienced interventional cardiologists examined the data from the coronary angiograms. CAD was confirmed by presence of coronary stenosis (≥ 50%) in one or more of the three main coronary arteries or their major branches (branch diameter ≥ 2 mm). To analyze the influence of hub gene on the prognosis of CAD, after coronary angiography, we divided the individuals into two groups, including CAD patients and controls. Patients with a previous CAD attack, type I diabetes mellitus and congenital heart disease were excluded [24]. The absence of CAD in healthy individuals was confirmed through clinical examination, medical history and questionnaires. The medical history and general information of all subjects were obtained by a standard questionnaire. All protocols followed the guidelines of the revised (in 2008) Declaration of Helsinki of 1975 (http://www.wma.net/en/30publications/10policies/b3/). Informed consent was obtained from all subjects involved in the genetic analysis and epidemiologic investigation. Our study was approved by the Ethics Committee of the First Affiliated Hospital, Guangxi Medical University (No: Lunshen-2011-KY-Guoji-001; Mar. 7, 2011) [25]. During the initial examination, clinical information was obtained from the medical records.

### Serum hub gene determination

RT-qPCR was used to validate the four significantly dysregulated mRNAs obtained from the microarray results out of the 206 researchers. The PBMCs extracted from blood samples were used to isolate total RNAs were using TRIzol reagent (Invitrogen). The TransScript R Frist-Strand cDNA Synthesis SuperMix (Transgen, China) was used to synthesize cDNA as per the manufacturer’s protocols. Additional file 1 Table S1 shows the sequences of primers used to probe the specific genes which were designed by Sangon Biotech (Shanghai, China) together with the reaction conditions. All samples were put into a 96-well plates and run in triplicate using the LightCycler R 96 and FastStart Essential DNA Green Master (Roche Diagnostics GmbH, Germany). The fit point method was used to calculate the Quantification cycles (Cq) by the LightCycler R 96 Software, (Version 1.1 provided by Roche). The mRNA levels of the genes were normalized to the expression of GAPDH as a reference. All methods (qPCR normalization, primer design, collection, preparation and storage of sample) were carried out in line with the MIQE guidelines.

**Table 1:** GO for differentially expressed genes

Category	ID	Description	GeneRatio	Adj-*P*	Gene ID
BP	GO:0001525	angiogenesis	35/260	4.58E-13	NR4A1/JUN/TNFAIP3/IL1B/PTGS2/EREG/BTG1/SHB/EPAS1/MMP2/GPNMB/CYR61/ENG/ITGAV/SERPINE1/HMOX1/MMP19/WNT5A/JAG1/CTGF/FN1/NRP1/VEGFA/LEF1/COL4A1/SULF1/ACKR3/CCL2/SPHK1/PLXDC1/ANGPTL4/FLT1/EMP2/ACVRL1/SLIT2
BP	GO:0048514	blood vessel morphogenesis	35/260	5.47E-11	NR4A1/JUN/TNFAIP3/IL1B/PTGS2/EREG/BTG1/SHB/EPAS1/MMP2/GPNMB/CYR61/ENG/ITGAV/SERPINE1/HMOX1/MMP19/WNT5A/JAG1/CTGF/FN1/NRP1/VEGFA/LEF1/COL4A1/SULF1/ACKR3/CCL2/SPHK1/PLXDC1/ANGPTL4/FLT1/EMP2/ACVRL1/SLIT2
BP	GO:0030335	positive regulation of cell migration	31/260	8.84E-11	JUN/HBEGF/PTGS2/CXCL2/ADAM9/POSTN/GPNMB/CYR61/ITGAV/SERPINE1/ICAM1/DOCK1/MET/CXCL12/CCL20/WNT5A/CXCL3/CCL7/FN1/NRP1/VEGFA/LEF1/PLPP3/ACKR3/CCL2/SPHK1/FLT1/CXCL16/KITLG/FOXP1/ITGA4
BP	GO:2000147	positive regulation of cell motility	31/260	1.47E-10	JUN/HBEGF/PTGS2/CXCL2/ADAM9/POSTN/GPNMB/CYR61/ITGAV/SERPINE1/ICAM1/DOCK1/MET/CXCL12/CCL20/WNT5A/CXCL3/CCL7/FN1/NRP1/VEGFA/LEF1/PLPP3/ACKR3/CCL2/SPHK1/FLT1/CXCL16/KITLG/FOXP1/ITGA4
BP	GO:0040017	positive regulation of locomotion	32/260	1.47E-10	JUN/HBEGF/PTGS2/CXCL2/ADAM9/POSTN/GPNMB/CYR61/ITGAV/SERPINE1/ICAM1/DOCK1/MET/CXCL12/CCL20/WNT5A/CXCL3/CCL7/FN1/NRP1/VEGFA/LEF1/PLPP3/ACKR3/CCL2/SPHK1/FLT1/CXCL16/KITLG/FOXP1/SLIT2/ITGA4
BP	GO:0051272	positive regulation of cellular component movement	31/260	1.99E-10	JUN/HBEGF/PTGS2/CXCL2/ADAM9/POSTN/GPNMB/CYR61/ITGAV/SERPINE1/ICAM1/DOCK1/MET/CXCL12/CCL20/WNT5A/CXCL3/CCL7/FN1/NRP1/VEGFA/LEF1/PLPP3/ACKR3/CCL2/SPHK1/FLT1/CXCL16/KITLG/FOXP1/ITGA4
BP	GO:0030198	extracellular matrix organization	27/260	1.99E-10	POSTN/SPP1/MMP2/HTRA1/BGN/CYR61/TIMP1/LUM/ENG/CTSL/ITGAV/SERPINE1/ICAM1/VCAM1/MMP19/MMP12/MMP10/P4HA1/CTGF/GPM6B/LAMB3/FN1/COL4A1/SULF1/JAM2/MELTF/ITGA4
BP	GO:0043062	extracellular structure organization	27/260	1.99E-10	POSTN/SPP1/MMP2/HTRA1/BGN/CYR61/TIMP1/LUM/ENG/CTSL/ITGAV/SERPINE1/ICAM1/VCAM1/MMP19/MMP12/MMP10/P4HA1/CTGF/GPM6B/LAMB3/FN1/COL4A1/SULF1/JAM2/MELTF/ITGA4
BP	GO:0050900	leukocyte migration	32/260	2.16E-10	TREM1/IL1B/CXCR4/CXCL2/SDC4/ITGAV/SERPINE1/ICAM1/SLC7A6/PROCR/HMOX1/CXCL12/VCAM1/CCL20/WNT5A/MERTK/CXCL3/CCL22/CCL7/OLR1/FN1/VEGFA/SDC2/FPR3/CCL2/JAM2/FLT1/CXCL16/KITLG/ATP1B3/SLIT2/ITGA4
BP	GO:0060326	cell chemotaxis	24/260	2.76E-10	NR4A1/TREM1/HBEGF/IL1B/CXCR4/CXCL2/RAB13/SERPINE1/MET/CXCL12/VCAM1/CCL20/WNT5A/CXCL3/CCL22/CCL7/NRP1/VEGFA/LEF1/FPR3/CCL2/FLT1/CXCL16/SLIT2
BP	GO:0042326	negative regulation of phosphorylation	29/260	7.69E-09	DUSP1/TRIB1/JUN/TNFAIP3/SOCS3/IL1B/DUSP2/CISH/DACT1/DDIT4/BGN/PPIF/ENG/WWTR1/TRIB2/GPRC5A/DUSP4/SMAD6/FBP1/INHBA/PLPP3/SPRED2/PKIB/ERRFI1/SPRED1/CNKSR3/SOCS6/SLIT2/TNIP1
BP	GO:0016049	cell growth	30/260	2.02E-08	SGK1/HBEGF/CISH/BTG1/POSTN/IGFBP5/SPP1/HTRA1/CYR61/EMP1/GJA1/ITGAV/CXCL12/WNT5A/PPARG/CTGF/FBP1/FN1/NRP1/INHBA/VEGFA/LEF1/EPB41L3/SPHK1/CXCL16/RAPH1/ACVRL1/TMC8/SLIT2/DCUN1D3
BP	GO:0001558	regulation of cell growth	26/260	4.66E-08	SGK1/HBEGF/CISH/BTG1/IGFBP5/SPP1/HTRA1/CYR61/GJA1/CXCL12/WNT5A/PPARG/CTGF/FBP1/FN1/NRP1/INHBA/VEGFA/LEF1/EPB41L3/SPHK1/CXCL16/ACVRL1/TMC8/SLIT2/DCUN1D3
BP	GO:0001933	negative regulation of protein phosphorylation	26/260	1.12E-07	DUSP1/TRIB1/JUN/TNFAIP3/SOCS3/IL1B/DUSP2/CISH/DACT1/DDIT4/BGN/ENG/WWTR1/TRIB2/GPRC5A/DUSP4/SMAD6/PLPP3/SPRED2/PKIB/ERRFI1/SPRED1/CNKSR3/SOCS6/SLIT2/TNIP1
BP	GO:0045785	positive regulation of cell adhesion	25/260	2.89E-07	IL1B/HLA-DRB5/CD83/CD80/ADAM9/CD59/TGM2/CYR61/MYO10/SDC4/ITGAV/ICAM1/DOCK1/CXCL12/VCAM1/WNT5A/NR4A3/FN1/VEGFA/PLPP3/CCL2/TESPA1/EMP2/IL6ST/ITGA4
BP	GO:0048660	regulation of smooth muscle cell proliferation	14/260	4.16E-07	TRIB1/JUN/TNFAIP3/HBEGF/PTGS2/EREG/NAMPT/IGFBP5/TGM2/MMP2/HMOX1/NR4A3/PPARG/FOXP1
BP	GO:0048659	smooth muscle cell proliferation	14/260	5.55E-07	TRIB1/JUN/TNFAIP3/HBEGF/PTGS2/EREG/NAMPT/IGFBP5/TGM2/MMP2/HMOX1/NR4A3/PPARG/FOXP1
BP	GO:0045765	regulation of angiogenesis	17/260	6.74E-06	TNFAIP3/IL1B/PTGS2/BTG1/GPNMB/ENG/SERPINE1/HMOX1/WNT5A/VEGFA/SULF1/CCL2/SPHK1/ANGPTL4/FLT1/EMP2/ACVRL1
BP	GO:0033002	muscle cell proliferation	15/260	6.74E-06	TRIB1/JUN/TNFAIP3/HBEGF/PTGS2/EREG/NAMPT/IGFBP5/TGM2/MMP2/GJA1/HMOX1/NR4A3/PPARG/FOXP1
BP	GO:0070098	chemokine-mediated signaling pathway	11/260	6.74E-06	CXCR4/CXCL2/CXCL12/CCL20/CXCL3/CCL22/CCL7/CCRL2/ACKR3/CCL2/SLIT2
BP	GO:0030595	leukocyte chemotaxis	16/260	6.74E-06	TREM1/IL1B/CXCR4/CXCL2/SERPINE1/CXCL12/CCL20/WNT5A/CXCL3/CCL22/CCL7/VEGFA/CCL2/FLT1/CXCL16/SLIT2
BP	GO:0032496	response to lipopolysaccharide	20/260	7.67E-06	TRIB1/JUN/TNFAIP3/IL1B/PTGS2/CXCL2/CD80/GNG12/ADAM9/GJA1/SERPINE1/ICAM1/VCAM1/MRC1/CCL20/WNT5A/CXCL3/CCL2/CXCL16/FOXP1
BP	GO:0018108	peptidyl-tyrosine phosphorylation	22/260	8.10E-06	SOCS3/HBEGF/OSM/EREG/CD80/TTN/EFEMP1/ICAM1/GPRC5A/MET/MERTK/TXK/NRP1/VEGFA/PLPP3/FLT1/ERRFI1/KITLG/EHD4/ABI2/ABL2/IL6ST
BP	GO:0018212	peptidyl-tyrosine modification	22/260	8.54E-06	SOCS3/HBEGF/OSM/EREG/CD80/TTN/EFEMP1/ICAM1/GPRC5A/MET/MERTK/TXK/NRP1/VEGFA/PLPP3/FLT1/ERRFI1/KITLG/EHD4/ABI2/ABL2/IL6ST
BP	GO:0001667	ameboidal-type cell migration	20/260	1.27E-05	NR4A1/JUN/HBEGF/PTGS2/ADAM9/TIMP1/SDC4/RAB13/DOCK1/MET/WNT5A/NRP1/VEGFA/LEF1/EMP2/KITLG/ACVRL1/FOXP1/SLIT2/ITGA4
BP	GO:0002237	response to molecule of bacterial origin	20/260	1.43E-05	TRIB1/JUN/TNFAIP3/IL1B/PTGS2/CXCL2/CD80/GNG12/ADAM9/GJA1/SERPINE1/ICAM1/VCAM1/MRC1/CCL20/WNT5A/CXCL3/CCL2/CXCL16/FOXP1
BP	GO:0001706	endoderm formation	9/260	1.49E-05	DUSP1/DUSP2/MMP2/ITGAV/DUSP4/LAMB3/FN1/INHBA/ITGA4
BP	GO:0022617	extracellular matrix disassembly	11/260	1.51E-05	SPP1/MMP2/HTRA1/TIMP1/CTSL/MMP19/MMP12/MMP10/LAMB3/FN1/MELTF
BP	GO:0070371	ERK1 and ERK2 cascade	18/260	1.51E-05	DUSP1/JUN/GPNMB/CYR61/ITGAV/ICAM1/DUSP4/CCL20/CCL22/CCL7/CTGF/FN1/NRP1/ACKR3/CCL2/ERRFI1/CNKSR3/TNIP1
BP	GO:0032963	collagen metabolic process	12/260	1.51E-05	MMP2/ENG/CTSL/MMP19/MMP12/MMP10/PPARG/CTGF/CIITA/COL4A1/CCL2/ERRFI1
BP	GO:0048661	positive regulation of smooth muscle cell proliferation	10/260	1.53E-05	JUN/HBEGF/PTGS2/EREG/NAMPT/TGM2/MMP2/HMOX1/NR4A3/FOXP1
BP	GO:0002685	regulation of leukocyte migration	14/260	1.58E-05	CXCL2/SERPINE1/ICAM1/HMOX1/CXCL12/CCL20/WNT5A/CXCL3/CCL7/VEGFA/CCL2/KITLG/SLIT2/ITGA4
BP	GO:0022604	regulation of cell morphogenesis	23/260	1.69E-05	RHOJ/POSTN/SH3D19/SPP1/MYO10/S100A13/ICAM1/DOCK1/CXCL12/WNT5A/SKIL/CCL7/FERMT2/FN1/NRP1/VEGFA/SDC2/EPB41L3/CSNK1D/CCL2/MELTF/ARHGAP18/SLIT2
BP	GO:1901342	regulation of vasculature development	17/260	1.69E-05	TNFAIP3/IL1B/PTGS2/BTG1/GPNMB/ENG/SERPINE1/HMOX1/WNT5A/VEGFA/SULF1/CCL2/SPHK1/ANGPTL4/FLT1/EMP2/ACVRL1
CC	GO:0005578	proteinaceous extracellular matrix	17/270	0.0098426	POSTN/MMP2/BGN/TIMP1/LUM/EFEMP1/MGP/MMP19/MMP12/MMP10/WNT5A/CTGF/LAMB3/FN1/VEGFA/COL4A1/ANGPTL4
MF	GO:0005126	cytokine receptor binding	20/264	2.31E-06	IL1B/OSM/CXCL2/ENG/CXCL12/CCL20/SMAD6/CXCL3/CCL22/CCL7/INHBA/VEGFA/CCRL2/SPRED2/CCL2/CXCL16/KITLG/SPRED1/TNFSF15/IL6ST
MF	GO:0005125	cytokine activity	18/264	2.31E-06	IL1B/OSM/NAMPT/CXCL2/SPP1/TIMP1/CXCL12/CCL20/WNT5A/CXCL3/CCL22/CCL7/INHBA/VEGFA/CCL2/CXCL16/KITLG/TNFSF15
MF	GO:0019838	growth factor binding	13/264	1.55E-05	DUSP1/IGFBP5/HTRA1/CYR61/ENG/ITGAV/S100A13/CTGF/NRP1/COL4A1/FLT1/ACVRL1/IL6ST
MF	GO:0001968	fibronectin binding	7/264	1.61E-05	IGFBP5/SDC4/CTSL/ITGAV/CTGF/VEGFA/ITGA4
MF	GO:0042379	chemokine receptor binding	9/264	2.36E-05	CXCL2/CXCL12/CCL20/CXCL3/CCL22/CCL7/CCRL2/CCL2/CXCL16
MF	GO:0008009	chemokine activity	8/264	5.25E-05	CXCL2/CXCL12/CCL20/CXCL3/CCL22/CCL7/CCL2/CXCL16

### Statistical analyses

SPSS 21.0 package (SPSS Inc. Chicago, IL, USA) was used for statistical analysis. Differences in the rates between groups were compared using a chi-square. Continuous data are presented as the means ± SD. Nominal significance was considered for a raw *P* value of < 0.05. Multivariate Cox proportional-hazards regression and univariate analyses were used to determine the correlation between the clinical variables and genes with the end point of cardiac adverse events. The univariate and multivariate tests were carried out using two-sided Cox univariate analyses.

## Results

### Preprocessing

After analysis of GSE19339 and GSE56885, from each gene expression and profile, we obtained a total of 54,560 expression probes. The preprocessed data are shown in Additional file 2 Figure S1. When all of the median values were in the same horizontal line, the data were comfortable normalized.

**Fig. 1:**
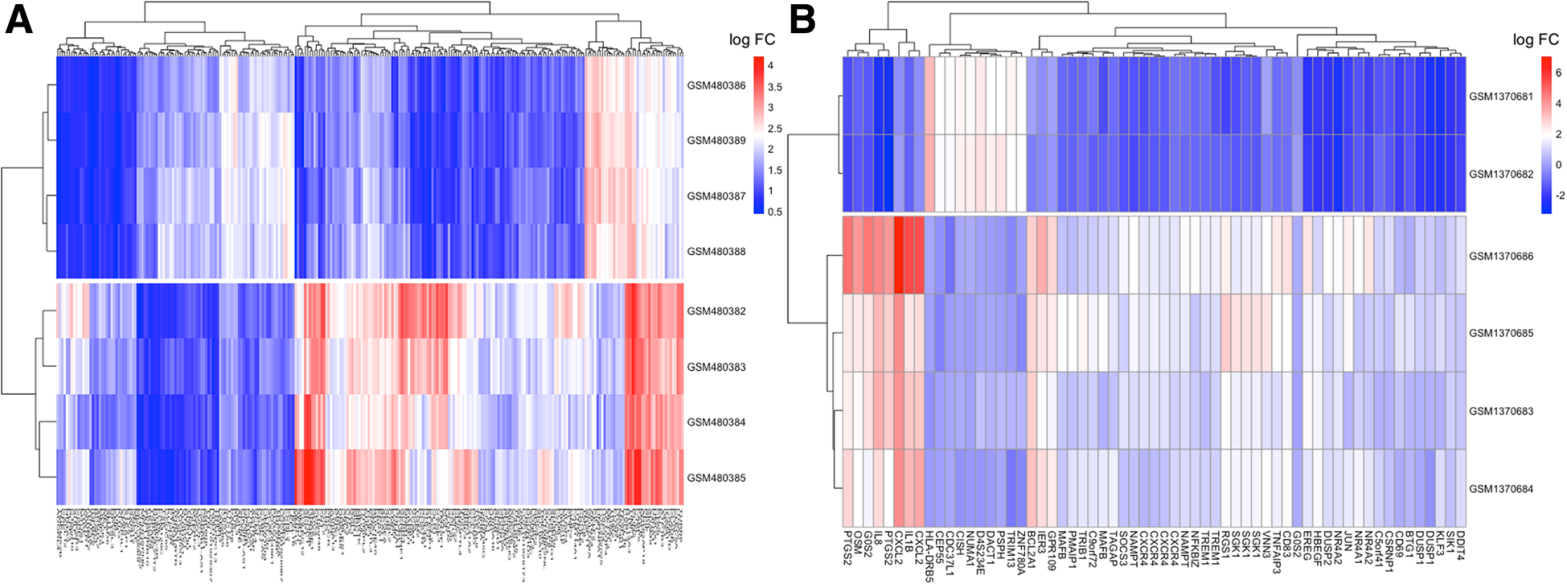
Heat map of differentially expressed genes. The depth of color reflects the level of differential expression (represents by log FC). **a** GSE19339; (**b**): GSE56885

### Identified differentially expressed genes (DEGs)

**Fig. 2:**
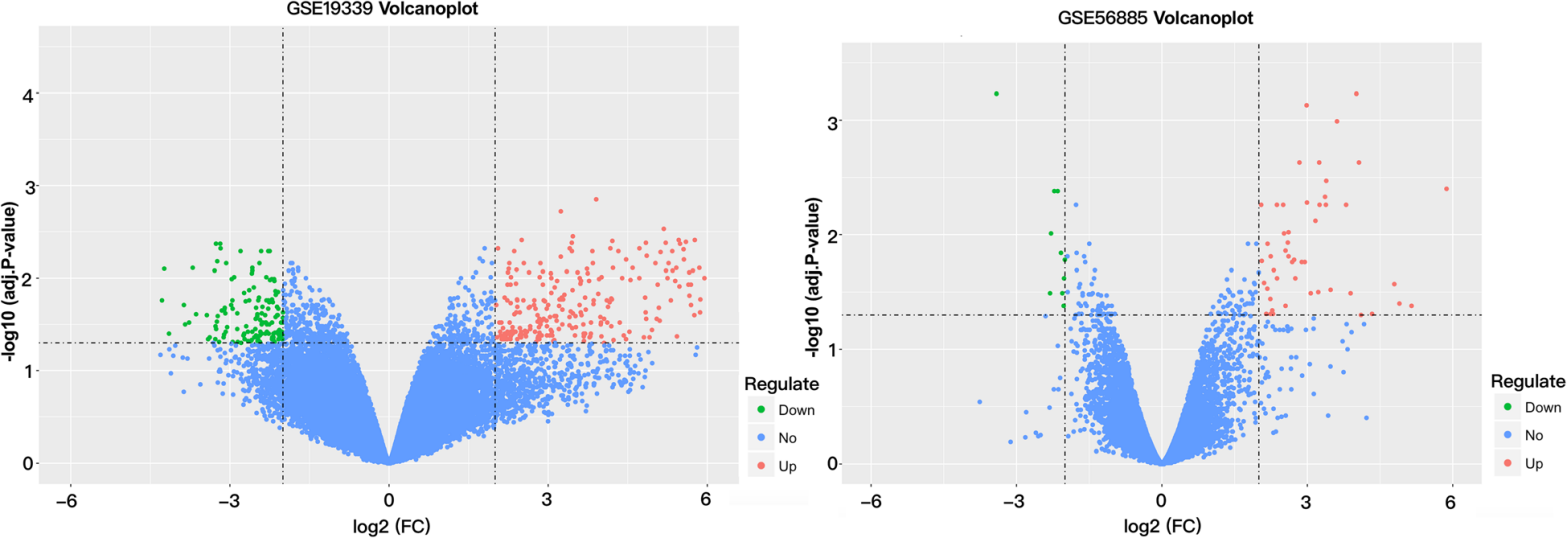
Identifying the DEGs by a volcano plot. The two vertical lines are the 2-fold change boundaries and the horizontal line is the statistical significance boundary (Adj-*P* < 0.05). Genes with a fold change ≥2 and a statistical significance and up-regulation are marked with red dots, and the down-regulated genes are marked with green dots. The horizontal line represents the boundary for statistical significance and the two vertical lines represent the boundaries of 2-fold change (Adj-*P* < 0.05). Red dots represent up-regulated and statistically significant genes with a fold change ≥2 and the down-regulated genes are marked with green dots

Heat map of differentially expressed genes is presented in Fig. 1. As shown in Fig. 2, with |log _2_ (fold change) | ≥ 2, and an adjusted-*P* value < 0.05. A sum of 402 DEGs were obtained, of which 140 were down-regulated while 262 were up-regulated in GSE19339. At the same time, 71 DEGs in GSE56885 were found: 10 genes were down-regulated and 61 were up-regulated. Some probes were too high or too low to be expressed. We have determined such probes as outliers and need to be removed without further analysis. In cases where many probes corresponded to one gene, we used the average expression value to screen for differential genes. After quality control and removing numerous incorrect expression values, we took all of the 413 DEGs (122 down-regulated and 291 up-regulated) into consideration.

### Analysis of gene ontology functions and KEGG pathways enrichment of DEGs

**Fig. 3:**
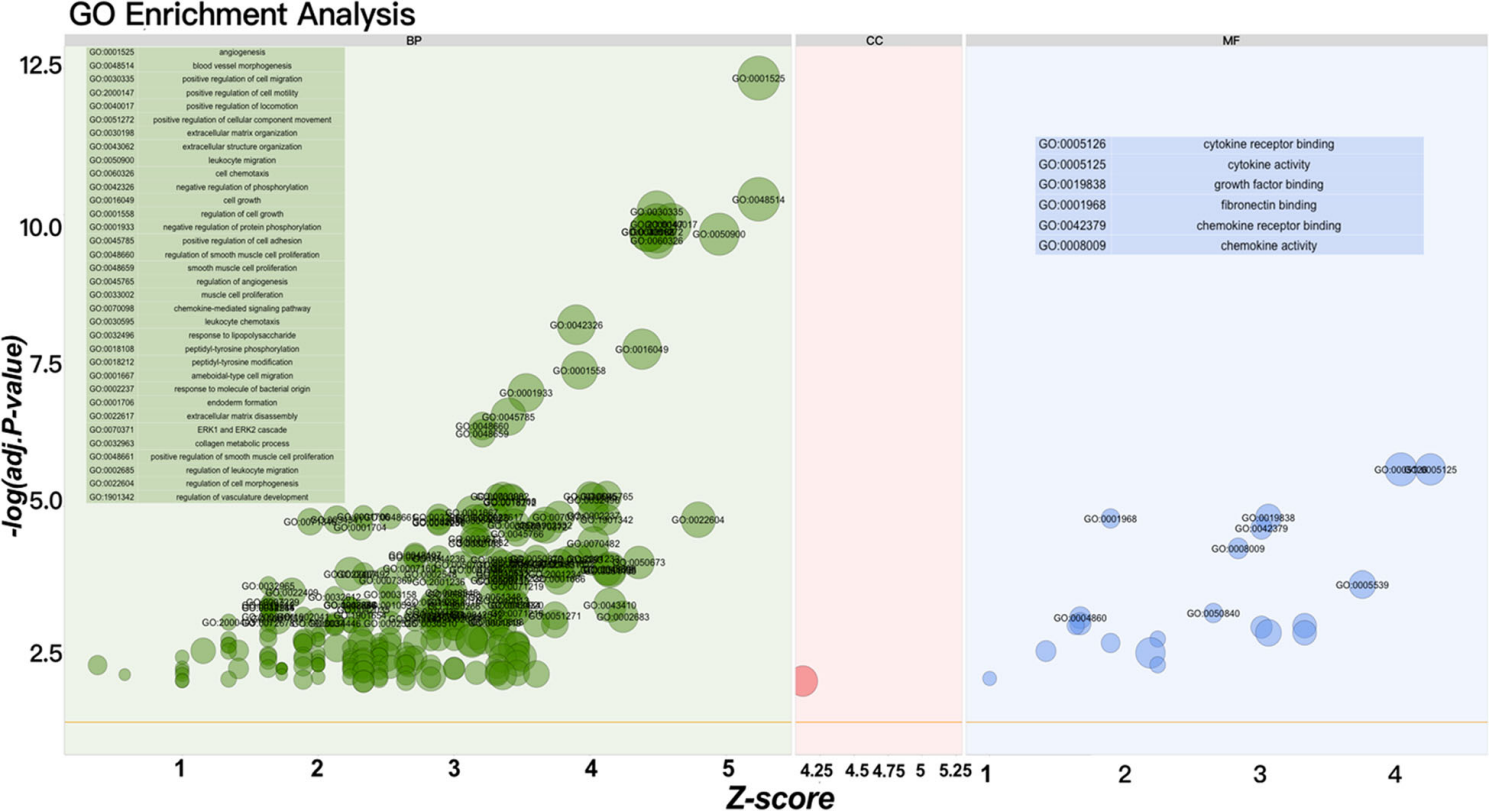
Functional enrichment analysis for the up-regulated DEGs. The y-axis indicates the identified Adj-*P* value. The x-axis represents the Z-score. The light green background represents the biological process, and 34 functional enrichments are also shown in this. The light blue background represents the molecular function, and 6 functional enrichments are also shown in this

**Fig. 4:**
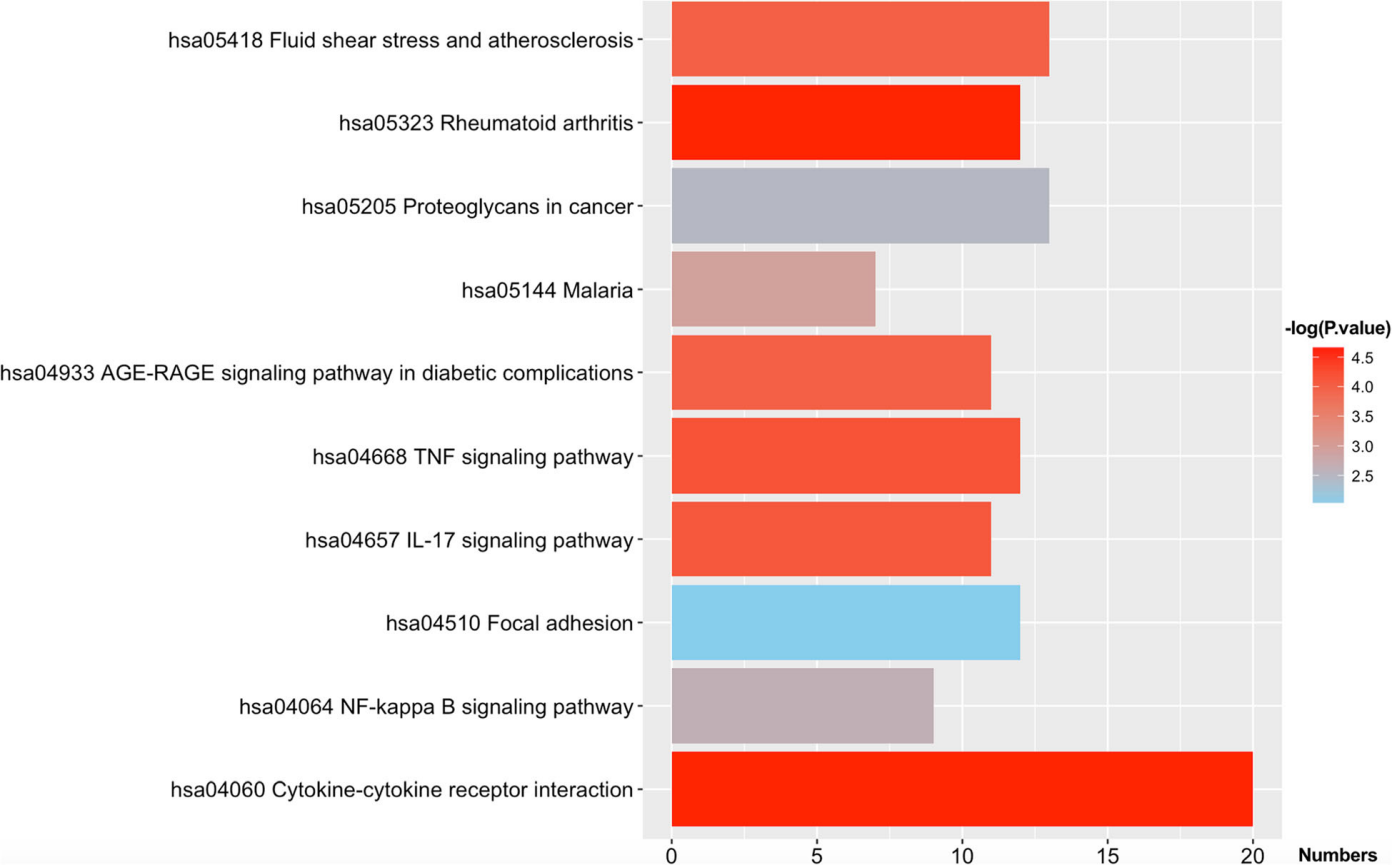
Results of KEGG pathway enrichment analysis for the DEGs

**Fig. 5:**
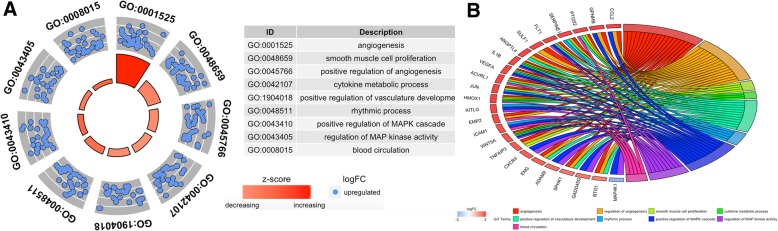
The enriched gene ontology terms and pathways of the differentially expressed genes (DEGs). **a** bar plot is shown in the inner ring, with the color corresponding to the z-score and the height of the bar representing the significance of the term. The scatter plots of the expression levels (logFC) of the genes in each term are shown in the outer ring. **b** ribbons linking the genes with their assigned terms. The logFC is represented by the blue-to-red coding near the marked genes

In the analysis of GO functions, 256 biological processes, only 1 cellular component, and 21 molecular functions were identified. All of these data are presented in Table 1. As shown in Fig. 3, if the statistics threshold was adjusted to 3, only 34 biological processes and 6 molecular functions were retained for analysis. From these data, we found that several functions were related to angiogenesis. The DAVID tool (version 6.8) was also used for the KEGG pathway analysis of the screened DEGs. Approximately 10 pathways were enriched (Fig. 4). To identify disease-related genes, analysis of KEGG pathways and GO functions was performed. A total of 24 genes (Fig. 5b) were enriched in 9 biological processes (Fig. 5a) and 7 KEGG pathways, including biological processes (angiogenesis, blood vessel morphogenesis, smooth muscle cell proliferation, positive regulation of angiogenesis, vasculature development, MAPK cascade, regulation of MAP kinase activity, blood circulation and rhythmic process) and KEGG pathway (atherosclerosis, rheumatoid arthritis, fluid shear stress, AGE-RAGE signaling pathway in diabetic complications, tumor necrosis factor (TNF), cytokine-cytokine receptor interaction, interleukin (IL)-17 and NF-kappa B signaling pathway).

### Protein-protein interaction (PPI) network construction

**Fig. 6:**
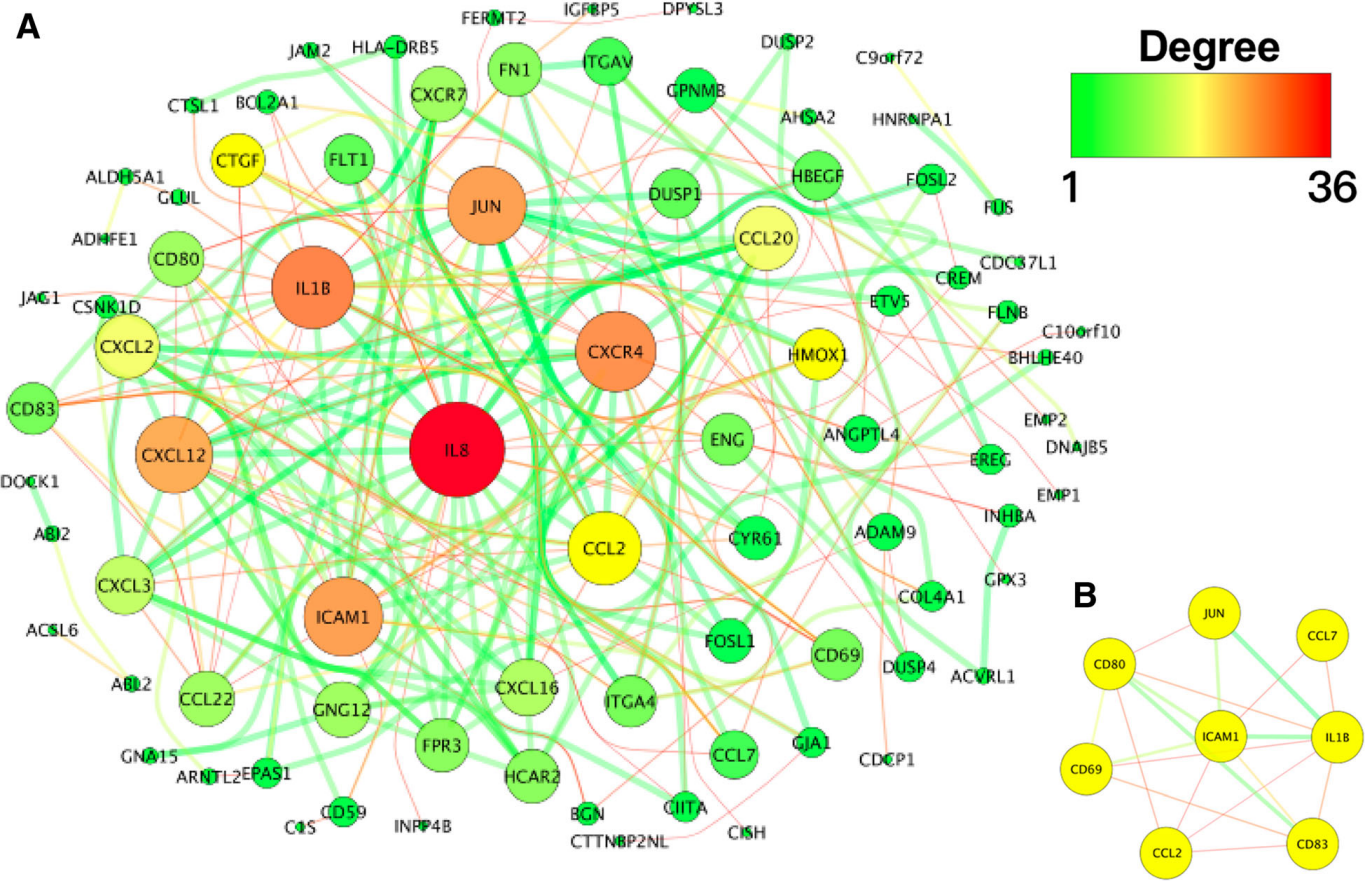
The protein-protein interaction analysis of the differentially expressed genes. **a** Protein–protein interaction network of the selected differentially expressed genes (DEGs). The edge indicates the interaction between two genes. A degree is used to describe the importance of the protein nodes in the network, red shows a high degree and green presents a low degree. **b** The significant modules identified from the protein-protein interaction network using the molecular complex detection method with a score > 6.0. MCODE score = 7.268

Data analysis was performed on the STRING database out of which 264 protein pairs and 64 nodes were revealed with a combined score > 0.9. The top 7 high degree genes, including interleukin 8 (*IL8*, degree = 34), interleukin 1 beta (*IL1B*, degree = 29), C-X-C motif chemokine receptor 4 (*CXCR4*, degree = 27), intercellular adhesion molecule 1 (*ICAM1*, degree = 25), Jun proto-oncogene (*JUN*, degree = 23), C-X-C motif chemokine ligand 12 (*CXCL12*, degree = 21) and C-C motif chemokine ligand 2 (*CCL2*, degree = 20), are shown in Fig. 6a.

### Module analysis

For the detection by MCODE with Cytoscape app, only one module with a score > 6 was found. As shown in Fig. 6b, the degrees of hub nodes of the *ICAM, IL1B, JUN* and *CCL2* were the highest in this module.

### Association of serum hub gene levels with CAD

**Fig. 7:**
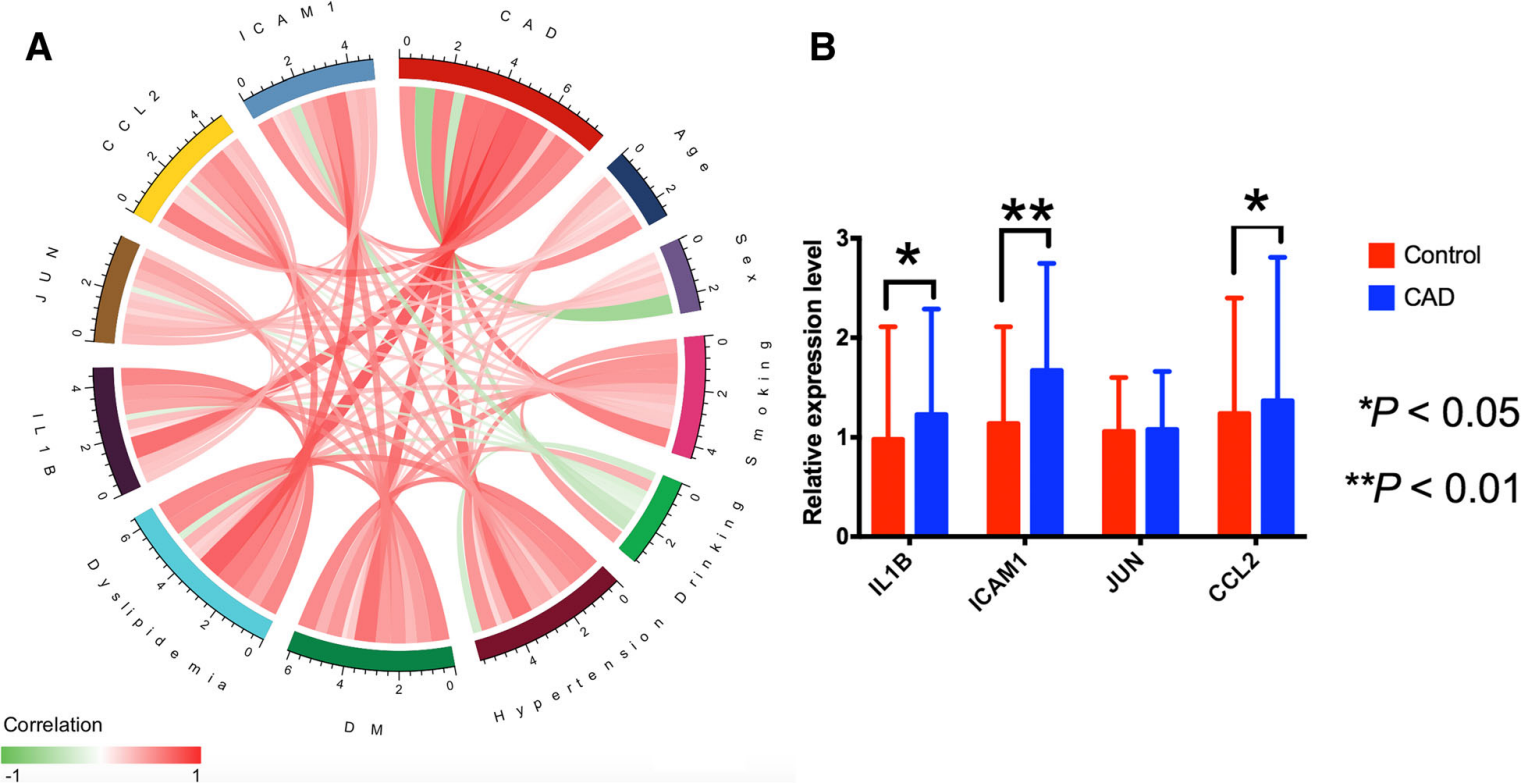
The relationship among CAD, genes and several environmental exposures and validation with RT-qPCR. **a** The associations among CAD, genes and environmental exposures. Positive correlation is marked with red, and negative correlation is marked with green. Scale represents the specific correlation coefficient. **b** An illustration of the expression profile of hub genes obtained from the microarray data verified by RT-qPCR. PBMCs were used to isolate total RNAs and subsequently cDNA for RT-qPCR analysis. The mRNA levels of each gene in healthy donors were considered as 1. *DM*, Diabetes Mellitus

Figure 7a shows the relationship among CAD, genes and environmental exposures and scale represents the specific correlation coefficient. Validation of the hypothesized data was done by RT-qPCR. The mRNA levels of *IL1B*, *JUN*, *ICAM1* and *CCL2* were determined to verify the major conclusions derived from the microarray results of the peripheral blood specimen. In general, results of the microarray analysis were consistent with those of RT-qPCR analysis. But, RT-qPCR results showed that the expression of *IL1B*, *ICAM1* and *CCL2* was higher in CAD patients than in normal controls (Fig. 7b).

### Demographic and cox regression analysis in patients with CAD

The demographic and biochemical characteristics of the participants in the two groups are presented in Table 2. Compared to CAD patients, there were more patients with hypertension and those who smoked cigarettes in the controls. But age, dyslipidemia, diabetes mellitus, alcohol drinking, height, weight, body mass index (BMI), waist circumference (WC), the level of systolic blood pressure (SBP), diastolic blood pressure (DBP), pulse pressure (PP), serum glucose, total cholesterol (TC), triglyceride (TG), low-density lipoprotein cholesterol (LDL-C) and high-density lipoprotein cholesterol (HDL-C) did not differ between controls and CAD patients. The predictors of CAD were further analyzed by univariate and multivariate Cox proportional hazards regression (Table 3). Following adjustment for variety clinical pathological factors and social economic factors, we confirmed that cigarette smoking [95% confidence interval (CI), 0.831–2.727, hazard ratio (HR): 1.506; *P* = 0.020], diabetes mellitus (95% CI, 1.634–8.283, *P* = 0.002 for HR: 3.679), and the overexpression of serum *IL1B* (95% CI, 1.435–2.845, *P* = 0.017 for HR: 1.896) and *CCL2* (95% CI, 0.563–2.440, *P* = 0.032 for HR: 1.172) genes were still considered independent predictors for CAD.

**Table 2:** Comparison of the demographics and lifestyle characteristics and the prevalence of related disease between the two groups

Parameter	CAD	Control	*test-statistic*	*P*
Number	110	96	–	–
Male/female	57.53 ± 12.51	58.22 ± 12.83	1.682	0.195
Age (years) ^a^	86/24	72/24	0.145	0.703
Height (cm)	155.64 ± 7.13	153.13 ± 6.93	1.495	0.181
Weight (kg)	60.71 ± 10.22	51.82 ± 7.94	24.419	1.65E-006
Body mass index (kg/m^2^)	31.44 ± 4.52	29.51 ± 3.22	29.224	1.97E-008
Waist circumference (cm)	84.55 ± 9.47	71.23 ± 6.91	20.321	2.98E-005
Smoking status [*n* (%)] ^b^	62(64.6)	36(32.7)	10.429	0.001
Alcohol consumption [*n* (%)]	68(70.8)	78(70.9)	0.009	0.993
Systolic blood pressure (mmHg)	136.47 ± 22.16	128.24 ± 18.18	43.136	6.13E-012
Diastolic blood pressure (mmHg)	86.49 ± 13.15	81.54 ± 10.16	18.250	7.39E-005
Pulse pressure (mmHg)	52.42 ± 17.59	49.64 ± 14.28	28.317	3.63E-07
Glucose (mmol/L)	7.15 ± 2.45	5.94 ± 1.83	19.817	5.91E-005
Total cholesterol (mmol/L)	5.14 ± 1.07	4.94 ± 1.13	7.121	0.029
Triglyceride (mmol/L) ^c^	1.78(1.22)	1.49(0.51)	8.441	0.021
HDL-C (mmol/L)	1.06 ± 0.27	1.51 ± 0.49	8.668	0.013
LDL-C (mmol/L)	2.88 ± 0.79	2.82 ± 0.84	9.497	0.007
ApoA1 (g/L)	1.29 ± 0.27	1.35 ± 0.25	0.364	0.558
ApoB (g/L)	0.86 ± 0.20	0.82 ± 0.19	1.492	0.233
ApoA1/ApoB	1.66 ± 0.57	1.67 ± 0.50	0.095	0.758
Dyslipidemia[n%]	60(62.5)	60(54.5)	0.667	0.414
Hypertension[n%]	82(85.4)	64(58.2)	9.201	0.002
Diabetes Mellitus[n%]	70(72.9)	72(65.5)	0.679	0.402

*HDL-C* high-density lipoprotein cholesterol, *LDL-C* low-density lipoprotein cholesterol, *Apo* Apolipoprotein

^a^Continuous data were presented as means ± SD and determined by two side *t*-test

^b^A chi-square analysis was used to evaluate the difference of the rate between the groups

^c^For those, that are normally distributed, whereas the medians and interquartile ranges for TG, was determined by the Wilcoxon-Mann-Whitney test

**Table 3:** Univariate and multivariate Cox hazards regression analysis of CAD

Parameter	Univariate HR (95% CI)	*P*-value	Multivariate HR (95% CI)	*P*-value
Age	1.003(0.984–1.022)	0.766	1.006(0.979–1.034)	0.660
Sex				
Male	1	–	1	–
Female	0.645(0.137–1.311)	0.225	0.442(0.145–1.351)	0.152
Cigarette Smoking				
No	1	–	1	–
Yes	1.506(0.831–2.727)	0.011	1.910(0.699–5.222)	0.020
Alcohol Drinking				
No	1	–	1	–
Yes	0.507(0.319–1.772)	0.043	0.986(0.580–2.805)	0.094
Dyslipidemia				
No	1	–	1	–
Yes	1.959(1.557–2.651)	0.023	1.831(1.375–2.842)	0.064
Hypertension				
No	1	–	1	–
Yes	1.914(1.085–3.375)	0.025	1.187(0.622–2.264)	0.060
Diabetes Mellitus				
No	1	–	1	–
Yes	1.970(1.103–3.518)	0.022	3.679(1.634–8.283)	0.002
IL1B	1.945(1.829–2.077)	0.009	1.896(1.435–2.845)	0.017
ICAM1	1.328(1.218–1.493)	0.087	1.134(1.010–2.758)	0.126
JUN	1.480(1.362–1.638)	0.059	1.528(1.270–5.652)	0.083
CCL2	1.945(0.830–2.088)	0.009	1.172(0.563–2.440)	0.032

*HR*, hazard rate; *CI*, confidence interval; *IL1B*, interleukin 1 beta; *ICAM1*, intercellular adhesion molecule 1; *JUN*, Jun proto-oncogene; *CCL2*, C-C motif chemokine ligand 2

## Discussion

With the remarkable improvement in microarray expression data, identifying abnormally expressed genes may help us to find and treat diseases. However, microarray data are not always reproducible or are too sensitive to errors [8]. With these situations, it may be a smart choice to remove the false positives by utilizing various datasets of parallel experimental designs. In the current study, we combined two different datasets of CAD to analyze their GO enrichments, KEGG pathways and PPI networks and modules to identify four significant and reproducible genes (*IL1B*, *ICAM1*, *JUN* and *CCL2*), which showed differential expression between the patients and controls. However, when these genes were replicated in our CAD samples, we found that serum *JUN* expression levels were not significantly changed and only two genes (*IL1B* and *CCL2*) were verified through the Cox proportional hazards regression.

CAD is a condition that is associated with several risk factors. The main pathophysiological mechanism of CAD is atherosclerosis [26]. Studies have confirmed that atherosclerosis is a chronic inflammatory disorder [27]. The IL-1 family, including cytokines, modulates many immunoinflammatory processes. It regulates many biological processes, including the lipoprotein metabolism, leukocyte adherence, thrombogenic response of endothelial cells, endothelial and smooth muscle cell monogenesis, vascular permeability and extracellular matrix production [28, 29]. It is also involved in the process of plaque formation and rupture via different pathways. The pathways’ function is summarized as follows: (1) the suppression of endothelial cell proliferation [30]; (2) the modification of the endothelium which later favors thrombosis [31]; (3) the stimulation of vascular smooth muscle cells via transforming growth factor-β (TGF-β) [32]; and (4) the expression of adhesion molecules by endothelial cells [33]. In the meantime, as a member of the IL-1 family of cytokines, ST2 (also known as T1, IL1RL1, or Fit1) measurements in blood samples could be a clinical prognostic biomarker useful in risk stratification of patients suffering from myocardial infarction, heart failure and dyspnea [34, 35]. In our current study, we demonstrated that *IL1B* participated in four of the main biological processes (Fig. 5b), including angiogenesis, smooth muscle cell proliferation, positive regulation of angiogenesis and cytokine metabolic process. All of these biological processes give rise to chronic immunoinflammatory pathological changes and finally result in atherosclerosis.

C-C motif chemokine ligand 2 (CCL2) participates in the genesis and progress of atherosclerosis [36–38]. The current study demonstrated that *CCL2* participated in two of the main biological processes (Fig. 5b), including angiogenesis and the positive regulation of angiogenesis. These two biological processes also contribute to atherosclerosis. Moreover, *CCL2* is considered as a risk factor for the promotion of atherosclerosis and for patients with CAD. Numerous studies reveal that high *CCL2* levels in patients with CAD are associated with enhanced incidence of adverse cardiac outcomes and increased risk of long-term mortality [39, 40]. Recently, a study showed when *CCL2* levels increased, plasma HDL2 levels decreased and *CCL2* was negatively correlated with HDL2 [41]. This may be another mechanism of how *CCL2* results in CAD.

There were seven pathways containing *IL1B* and *CCL2,* according to the KEGG analysis, including rheumatoid arthritis, cytokine-cytokine receptor interaction, TNF signaling pathway, the IL-17 signaling pathway, the AGE-RAGE signaling pathway in diabetic complications and malaria, atherosclerosis and fluid shear stress. Shanmugam et al. reported that TNF-α signaling exerted adverse effects to the cardiovascular tissues although it ameliorated chronic inflammatory disease [42]. In atherosclerosis, Tuenter et al. found that presence of intraplaque haemorrhage and calcifications was associated with elevated maximum shear stress [43]. Moreover, RAGE expression in many types of cell, including smooth muscle cells, endothelial cells and macrophages may result in the pathogenesis of atherosclerosis, and give rise to the pathogenesis of myocardial dysfunction [44]. These findings demonstrate that *IL1B* and *CCL2* cause atherosclerosis and finally result in CAD. Furthermore, when we repeated the validation in our experimental samples, using a blood gene expression and/or Cox proportional hazards regression, we obtained the same results, which might increase the credibility of the conclusions.

## Conclusions

Two CAD microarray datasets from the GEO series were systematically analyzed in this study. Based on the expression level, GO enrichment, enriched pathway and protein-protein interaction analyses, four genes (*IL1B, ICAM1, JUN* and *CCL2*) were found to be significant meaning, but only two genes (*IL1B* and *CCL2*) were replicated in our samples by testing the gene expression in blood and/or analyzing with a Cox proportional hazards regression. The mechanism may be involved in the cytokine-cytokine receptor interaction, the TNF signaling pathway, fluid shear stress and the AGE-RAGE signaling pathway. But, additional experiments are warranted to validate these findings.

## Additional files

Additional file 1: **Table S1.** PCR primers for quantitative real-time PCR. (PDF 70 kb)

Additional file 2: **Figure S1.** Box figure of gene expression data of normalization. (PDF 703 kb)

## Abbreviations

Apo: Apolipoprotein
BMI: Body mass index
COL1A1: Collagen type I alpha 1 chain
DBP: Diastolic blood pressure
GEO: Gene Expression Omnibus
GO: Gene Ontology annotation
HDL-C: High-density lipoprotein cholesterol
HTG: Hypertriglyceridemia
KEGG: Kyoto Encyclopedia of Genes and Genomes pathway enrichment analyses
LDL-C: Low-density lipoprotein cholesterol
MCODE: Molecular Complex Detection
MetS: Metabolic syndrome
NCBI: National Center For Biotechnology Information
PP: Pulse pressure
PPI: Protein-protein interaction
SBP: Systolic blood pressure
SNP: Single nucleotide polymorphism
T2D: Type 2 diabetes mellitus
TG: Triglyceride
WC: Waist circumference
