# Supplementary material for: Integrated analysis of gene expression changes associated with coronary artery disease
Source: Lipids Health Dis. 2019 Apr 9;18:92. doi: 10.1186/s12944-019-1032-5 (PMC6454774; doi:10.1186/s12944-019-1032-5)
Supplement: Supplementary file 1 — Table S1. PCR primers for quantitative real-time PCR. (PDF 70 kb) [file 12944_2019_1032_MOESM1_ESM.pdf]

Integrated analysis of gene expression changes associated with coronary artery disease

Liu Miao[1], Rui-Xing Yin[1], Feng Huang[1], Shuo Yang[1], Wu-Xian Chen[1], Jin-Zhen Wu[1]

[1] Department of Cardiology, Institute of Cardiovascular Diseases, The First Affiliated Hospital, Guangxi Medical University, Nanning 530021, Guangxi, People's Republic of China.

Running title: gene expression changes and coronary artery disease

Correspondence and requests for materials should be addressed to R.-X.Y. (email: yinruixing@163.com)

dr.miaoliu@qq.com
yinruixing@163.com
huangfeng3000@126.com
yangshuo1112@outlook.com
nncwx@163.com
wujianzhengx@sohu.com

Gene	Forward primer	Reverse primer
IL1B	AGTGGCACTGCAGGATCAAA	TTTACAGTTTTTGCCCGCGT
JUN	CTTTTTCGGCACTTGGAGG	GTCCGAGAGCGGACCTTATG
ICAM1	ACTGTGTGCCTATTCCAGGC	GGCTCACTCACAGAGCACAT
CCL2	ATGGACCATCCAAGCAGACG	CCCTTGCTCCACAAGGAAGA
GAPDH	AGAGAGAGGCCCTCAGTTGCT	TTGTGAGGGAGATGCTCAGTGT

Supplementary Table 1. PCR primers for quantitative real-time PCR.

It is OK!
Rui-Xing Yin
Mar. 2, 2019
