# Supplementary material for: Integrated analysis of gene expression changes associated with coronary artery disease
Source: Lipids Health Dis. 2019 Apr 9;18:92. doi: 10.1186/s12944-019-1032-5 (PMC6454774; doi:10.1186/s12944-019-1032-5)
Supplement: Supplementary file 2 — Figure S1. Box figure of gene expression data of normalization. (PDF 703 kb) [file 12944_2019_1032_MOESM2_ESM.pdf]

Integrated analysis of gene expression changes associated with coronary artery disease

Liu Miao[1], Rui-Xing Yin[1], Feng Huang[1], Shuo Yang[1], Wu-Xian Chen[1], Jin-Zhen Wu[1]

[1] Department of Cardiology, Institute of Cardiovascular Diseases, The First Affiliated Hospital, Guangxi Medical University, Nanning 530021, Guangxi, People's Republic of China.

Running title: gene expression changes and coronary artery disease

Correspondence and requests for materials should be addressed to R.-X.Y. (email: yinruixing@163.com)

dr.miaoliu@qq.com
yinruixing@163.com
huangfeng3000@126.com
yangshuo1112@outlook.com
nncwx@163.com
wujianzhengx@sohu.com

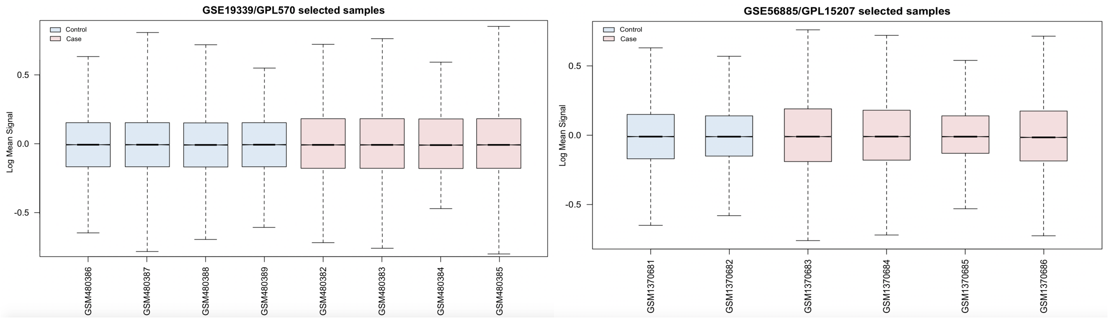

Supplemental Figure 1. Box figure of gene expression data of normalization. The horizontal axis represents the sample and the vertical axis represents the expression value. Black lines indicate the median values. Blue represents the control samples and red represents the CAD samples. The vertical axes represent expression signal values with log mean signals.

It is OK!
Rui-Xing Yin
Mar. 2, 2019
